# Delineating postinfarct ventricular tachycardia substrate with dynamic voltage mapping in areas of omnipolar vector disarray

**DOI:** 10.1016/j.hroo.2024.02.006

**Published:** 2024-02-27

**Authors:** Joao Grade Santos, Mark T. Mills, Peter Calvert, Nicole Worthington, Calum Phenton, Simon Modi, Reza Ashrafi, Derick Todd, Johan Waktare, Saagar Mahida, Dhiraj Gupta, Vishal Luther

**Affiliations:** ∗ Department of Cardiology, Liverpool Heart and Chest Hospital, Liverpool, United Kingdom; † Department of Cardiology, Hospital Garcia de Orta, Almada, Portugal; ‡ Liverpool Centre for Cardiovascular Science, University of Liverpool, Liverpool, United Kingdom; § Abbott Medical UK Ltd, Solihull, United Kingdom

**Keywords:** Ventricular tachycardia, Postinfarct scar, 3D mapping, Omnipolar electrograms, Activation vector, Ablation

## Abstract

**Background:**

Defining postinfarct ventricular arrhythmic substrate is challenging with voltage mapping alone, though it may be improved in combination with an activation map. Omnipolar technology on the EnSite X system displays activation as vectors that can be superimposed onto a voltage map.

**Objective:**

The study sought to optimize voltage map settings during ventricular tachycardia (VT) ablation, adjusting them dynamically using omnipolar vectors.

**Methods:**

Consecutive patients undergoing substrate mapping were retrospectively studied. We categorized omnipolar vectors as uniform when pointing in one direction, or in disarray when pointing in multiple directions. We superimposed vectors onto voltage maps colored purple in tissue >1.5 mV, and the voltage settings were adjusted so that uniform vectors appeared within purple voltages, a process termed dynamic voltage mapping (DVM). Vectors in disarray appeared within red-blue lower voltages.

**Results:**

A total of 17 substrate maps were studied in 14 patients (mean age 63 ± 13 years; mean left ventricular ejection fraction 35 ± 6%, median 4 [interquartile range 2-8.5] recent VT episodes). The DVM mean voltage threshold that differentiated tissue supporting uniform vectors from disarray was 0.27 mV, ranging between patients from 0.18 to 0.50 mV, with good interobserver agreement (median difference: 0.00 mV). We found that VT isthmus components, as well as sites of latest activation, isochronal crowding, and excellent pace maps colocated with tissue along the DVM border zone surrounding areas of disarray.

**Conclusion:**

DVM, guided by areas of omnipolar vector disarray, allows for individualized postinfarct ventricular substrate characterization. Tissue bordering areas of disarray may harbor greater arrhythmogenic potential.

**Key Findings**

▪ Omnipolar technology on the EnSite X system can display localized activation as vectors that can be superimposed onto a voltage map.
▪ In low-voltage tissue, we found that these vectors can appear in disarray, pointing in multiple directions.
▪ We describe an alternative approach to analyze a ventricular tachycardia substrate map, known as dynamic voltage mapping, adjusting the voltage display based on the location of vector disarray.
▪ In a small offline series of postinfarct scars, we found that ventricular tachycardia isthmus components, as well as sites of latest activation, isochronal crowding, and excellent pace maps colocated with low-voltage tissue bordering areas of disarray.
▪ The mean voltage differentiating tissue supporting uniform vectors from disarray was 0.27 mV, though it ranged widely, suggesting that there is no empirical voltage cutoff to analyze a ventricular tachycardia substrate map, and it must be individualized.

## Introduction

Postinfarct re-entrant ventricular tachycardia (VT) is dependent on regions of nonuniform conduction that reside within and around the infarct scar.^1^ As VT is often poorly tolerated, substrate mapping techniques have evolved to identify scar and its border zone in sinus or paced rhythms.^2^ Traditionally, scar has been identified as regions of low voltage (≤0.5 mV) depicted on a bipolar voltage map.^3^ However, studies have demonstrated viable tissue above 0.1 mV and considered variable voltage thresholds between 0.1 and 0.5 mV to improve substrate characterization.^4^^,^^5^ We recently described a stepwise approach to improve the utility of a bipolar voltage map during VT substrate ablation, by overlaying an activation map (CARTO Ripple Map; Biosense Webster, Diamond Bar, CA) onto the voltage display, and adjusting the voltage settings based on the pattern of activation observed.^6^ However, this study was limited to using bipolar electrograms (EGMs), which can change depending upon the wavefront direction relative to the recording electrodes.^4^ Omnipolar EGMs (Omni-EGMs) are wavefront independent and may better represent ventricular substrate.^7^^,^^8^ Omnipolar mapping has been integrated with the EnSite X platform (Abbott, Abbott Park, IL), demonstrating increased voltages compared with bipolar mapping within ventricular scar.^9^^,^^10^ Omnipolar activation can be displayed as a vector, represented by an arrow, which can be superimposed onto an omnipolar voltage map.

In this study, we describe an approach to help optimize the utility of a voltage map using omnipolar technology, which we term dynamic voltage mapping (DVM). We retrospectively apply this approach in a series of postinfarct VT substrate maps to better characterize the arrhythmic substrate.

## Methods

Consecutive patients with postinfarct scar who underwent VT substrate ablation using EnSite X at our institution (Liverpool Heart and Chest Hospital NHS Foundation Trust, Liverpool, United Kingdom) were studied. This platform includes omnipolar technology as a standard feature. Ablation procedures were clinically indicated for recurrent VT ± implantable cardioverter-defibrillator therapies (antitachycardia pacing or shocks). The study was approved by our local research committee and all patients gave informed consent for their clinical procedure. The study conforms to the principles outlined in the Declaration of Helsinki.

### Procedural details

Mapping was performed using the Advisor HD Grid mapping catheter (Abbott) that consists of 16 equally spaced electrodes (each 1 mm in size with 3-mm spacing) organized along 4 longitudinal splines. Automated omnipolar substrate maps were acquired in intrinsic rhythm, atrial pacing, or ventricular pacing (right ventricular apex, or basal lateral left ventricle via the coronary sinus or great cardiac vein) as per operator choice. Omnipolar voltage maps were displayed at traditional settings, with tissue showing peak-peak EGM voltage >1.5 mV (healthy tissue) colored purple, and tissue below this (diseased tissue) as per the rainbow color spectrum between red (lowest voltage) to blue. Activation timing maps were measured using the last deflection algorithm of the local EGM to better define areas of latest activation. Electroanatomical maps were also collected in tolerated VT after programmed stimulation. Interpolation around each data point was set to the default value of a 7-mm radius, and tissue was left uncolored where the interpoint distance exceeded this threshold.

Radiofrequency ablation was delivered between 30 and 50 W (titrated based on the rate of impedance drop), for a maximum 60 seconds, with a target contact force of 10–20 g, and aiming for an impedance drop of ideally 10–15 Ω. Conventional substrates associated with VT isthmus sites were targeted for ablation (TactiCath or TactiFlex catheter; Abbott) including (1) local abnormal ventricular activity, (2) areas of isolated late activation in areas of lower voltage, (3) decelerations zones within areas of isochronal crowding, and (4) 12/12 pace maps to any poorly tolerated or nonsustained VT in areas with prolonged stimulus-to-QRS interval.

Ablation during stable VT targeted diastolic activation. Programmed ventricular stimulation was used to guide acute ablation efficacy (600- and 400-ms drive trains with up to 3 extrastimuli down to ventricular refractoriness or 200 ms). All patients were monitored through their devices (in-house and remote) and followed-up in scheduled 3- to 6-monthly clinic appointments.

### Offline DVM approach

An offline analysis was conducted in each case to evaluate the omnipolar maps, the derivation of which has been previously described.^7^ In brief, omnipolar voltage is determined from triangular cliques of 3 adjacent electrodes on the HD Grid and uses advanced signal processing to calculate EGMs at each clique in 360°, selecting the Omni-EGM as that with the maximum peak-peak voltage for display on the omnipolar voltage map. Omnipolar vectors are derived by calculating the Omni-EGM with the greatest amplitude and referencing this EGM against the 3 unipolar electrodes within the triangular clique, allowing for directionality to be derived. This is displayed at a given location with an arrow.

Omnipolar vectors were displayed on each substrate map, and an Omnipolar Technology (OT) certainty threshold was applied to remove vectors in which the system was less certain of the calculated direction. There is limited data on the optimal setting for this threshold (0 = accept all level of uncertainty, incrementing by 0.1, up to 0.9 = display only vectors acquired with absolute levels of certainty). The default setting on EnSite X is 0.3. Our assessment of omnipolar vectors was impaired by low arrow density at 0.3, which also removed sharp fractionated Omni-EGMs from the map. For this study, we chose 0.1, to exclude vectors with only the greatest degree of system uncertainty that might remain at 0.0.

We observed that at any one time, a group of vectors can appear uniform when pointing in the same direction, or appear chaotic and unpredictable, which we described as vector disarray.^11^ We analyzed areas of vector disarray on the associated omnipolar voltage map and dynamically adjusted the voltage settings according to the location of vector disarray. We termed this approach DVM, which includes the following steps:

1. The upper voltage limit was reduced to 0.50 mV, displaying tissue >0.50 mV in purple and tissue <0.50 mV in nonpurple (using a blue-to-red rainbow color spectrum). While convention is to assign a lower voltage boundary to reflect dense scar, as the system hides any associated vectors below the lower voltage boundary (coloring the tissue in gray), this was set to 0 mV.
2. A line was drawn around tissue bordering 0.50 mV, highlighting tissue considered dense scar by traditional criteria.^3^
3. Omnipolar vectors were superimposed on the omnipolar voltage map. This allowed the vectors to be carefully studied together with the voltage map. The upper voltage limit was reduced until only those areas with vector disarray appeared nonpurple as diseased tissue. This voltage parameter was termed the DVM threshold (DVM-Th) (measured in mV).
4. Tissue immediately surrounding areas of vector disarray was classified as the DVM border zone.

**Figure 1:**
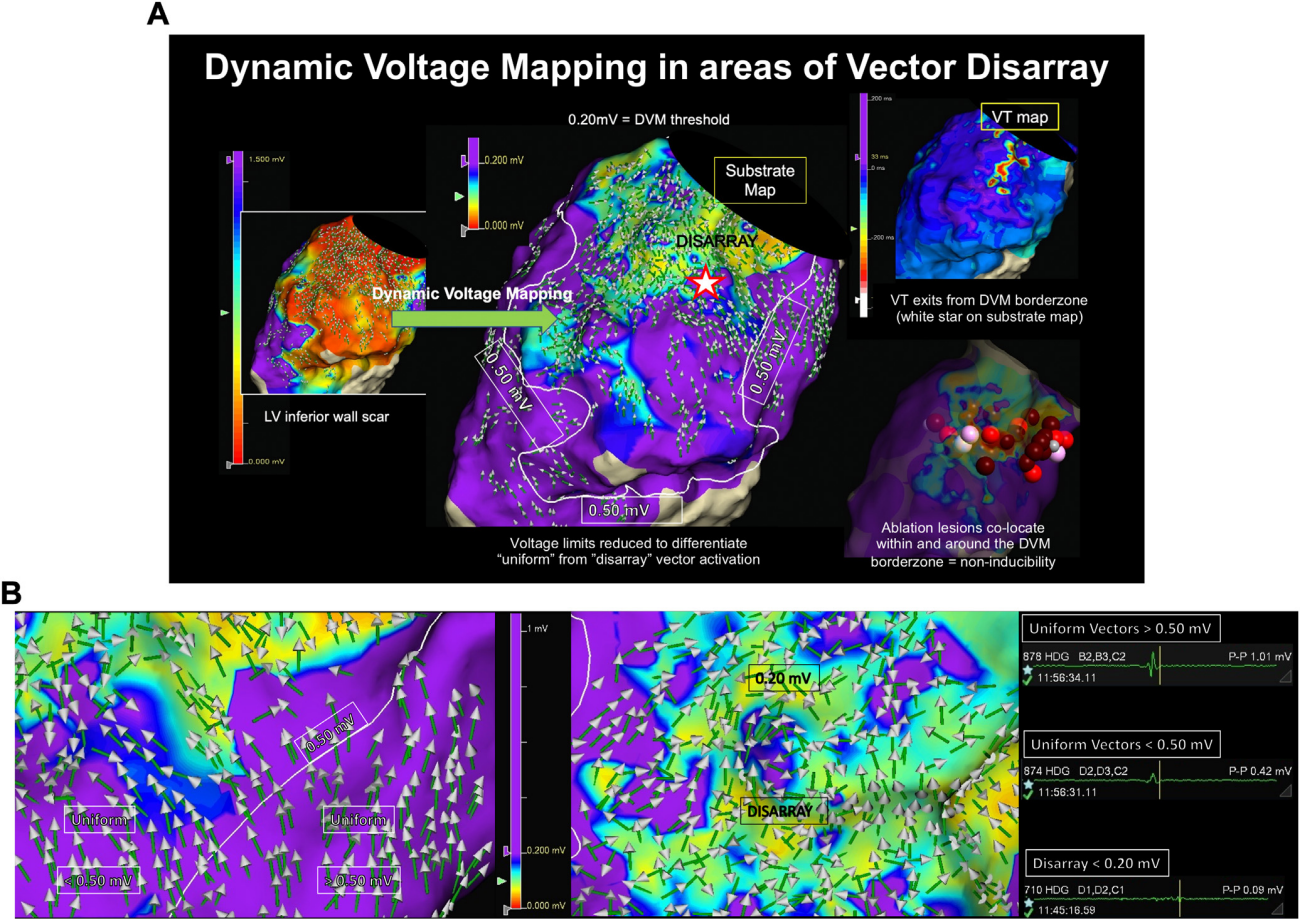
A: Dynamic voltage mapping (DVM) guided by omnipolar vector disarray (see also Video 1). (Left) Right ventricular–paced omnipolar voltage map of a left ventricular (LV) inferior wall scar displayed between 0.0 and 1.50 mV scale. Omnipolar vectors have been superimposed onto the voltage map. The upper voltage limit was subsequently adjusted to 0.50 mV and a line was drawn around tissue with voltage bordering 0.50 mV. (Middle) The voltage upper limit was sequentially reduced until only those areas with vector disarray were observed below it (a zoomed-in version is depicted in panel B). This voltage cutoff, termed the DVM threshold, here is 0.20 mV. (Right, top) Monomorphic ventricular tachycardia (VT) was mapped using local activation time—while the full circuit was not contained within the endocardial surface (evident by a color timing gap), components of the VT isthmus (red color) correlated with tissue bordering the DVM threshold. (Right, bottom) Ablation set colocating within and around the border zone of the DVM. B: Omnipolar vector uniformity and disarray (see also Video 1). (Left) Vector uniformity was observed above and below 0.50 mV (white line) in purple voltage tissue until the DVM threshold. (Middle) Zoomed area demonstrating vector disarray below the DVM threshold of 0.20 mV in tissue colored nonpurple (between red-blue). (Right, top) Electrogram from tissue above 0.50 mV. (Right, middle) Electrogram from tissue below 0.50 mV but with uniform activation. (Right, bottom) Electrogram from tissue below the DVM threshold.

This approach is exemplified in Figure 1A and Video 1. Figure 1B illustrates a magnified appearance of omnipolar vectors as both in uniform and in disarray.

### Offline DVM analytics

First, where there was uncertainty in classifying vector orientation, we arbitrarily chose to define an area of tissue as in disarray when the percentage uniformity was <50% based on the following formula:

$$Vector\;uniformity=\frac{\text{Number}\;\text{of}\;\text{vectors}\;\text{in}\;\text{the}\;\text{dominant}\;\text{direction}}{\text{Total}\;\text{number}\;\text{of}\;\text{vectors}}\;x\;100$$

Second, all maps were reviewed independently by 2 observers blinded to the other operator’s measurements, and for each DVM, we assessed the interobserver agreement of DVM-Th measurement.

Third, we analyzed the distribution of substrate ablation lesions in relation to the DVM border zone as follows:

$$=\frac{\begin{array}{c} \text{Number}\;\text{of}\;\text{ablation}\;\text{lesions}\;\text{delivered}\;\text{within}\;\text{or}\;\\ \;0.5\;\text{cm}\;\text{radius}\;\text{around}\;\text{the}\;\text{DVM}\;\text{border}\;\text{zone}\; \end{array}}{\text{Total}\;\text{number}\;\text{of}\;\text{ablation}\;\text{lesions}}\;x\;100$$

### Statistical analysis

Categorical variables are expressed as counts and percentages and continuous variables as mean ± 1 SD for parametric data or median (interquartile range) for nonparametric data. The data were analyzed using *t* tests for parametric data and Mann-Whitney *U* tests for nonparametric data. Paired data were analyzed using the paired *t* test or Wilcoxon signed rank test. Correlation was measured using Spearman’s rho for nonparametric data. A Bland-Altman plot was created to assess interobserver agreement in DVM-Th. Statistical analyses were performed in IBM SPSS Statistics, version 27.0.1.0. *P* values <.05 were considered statistically significant.

## Results

### Baseline characteristics

Fourteen patients were studied (Table 1). There was a male preponderance (12 patients) and a mean age of 63 ± 13 years. The time elapsed between infarct and presenting VT event was 19 ± 8 years. The mean left ventricular ejection fraction was 35 ± 6%. Ablation was performed for monomorphic VT and/or recurrent implantable cardioverter-defibrillator therapies (median 4 [interquartile range 2–8.5]) in the prior 3 months. Ten patients were on antiarrhythmic drugs, predominantly amiodarone and sotalol, and 3 had prior VT ablations.

**Table 1:** Patient characteristics, mapping, and ablation data

Patient	Age (y)	Sex	LVEF (%)	Prior 3-mo VT Burden (Episodes)	AAD	Prior VT ablation	Scar location	VT Inducible	Points	Bipolar Area <0.5 mV (cm^2^)	Omnipolar Area <0.5 mV (cm^2^)	Dynamic Voltage Map Threshold (mV)	Vector Disarray Area (cm^2^)	Number of Ablation Lesions	Ablation Duration (min)	DVM/Border Zone Ablation(%)	Postablation VT Inducible?
1	77	Male	33	2	Amiodarone	No	Inferior	Yes (n = 1)	3495	14.0	19.0	0.28	1.9	40	17	100	No
2	57	Male	33	2	None	No	Inferior	No	3172 2177	45.0 40.4	45.4 39.8	0.28 0.28	19.0 19.2	55	28	84	No
3	62	Male	25	3	Sotalol	Yes	Inferolateral	Yes (n = 1)	3374	54.0	48.8	0.25	11.3	34	19	88	No
4	65	Female	45	4	Sotalol	No	Lateral	Yes (n = 1)	1635	14.4	12.3	0.26	3.1	57	20	86	No
5	65	Male	35	12	Sotalol + mexiletine	No	Apical	No	9183∗	79.8∗	73.2∗	0.18∗	43.3∗	47	18	91	No
6	72	Male	34	2	Amiodarone	No	Inferior	Yes (n = 1)	5549	25.5	18.5	0.25	13.2	41	21	63	No
7	74	Male	32	50	Amiodarone	No	Apical	Yes (n = 5)	3069	10.5	7.7	0.45	5.8	113	51	84	No
8	51	Male	45	40	Amiodarone	No	Inferior	No	2923	53.2	45.4	0.20	34	67	18	87	No
9	76	Male	32	7	Amiodarone	No	Inferior	Yes (n = 2)	4791	36.7	33.5	0.25	20.4	74	44	95	No
10	47	Male	35	6	Sotalol	Yes	Inferior	Yes (n = 5)	1645	24.7	23.2	0.20	13.6	26	20	100	No
11	73	Male	33	3	None	No	Inferior	Yes (n = 4)	2921 4921	36.6 46.3	35.8 41.4	0.25 0.25	11.2 14.0	70	28	63	No
12	31	Female	30	0	None	No	Antero-septal	Yes (n = 1)	3856	59.5	55.1	0.26	18.5	39	26	23	Yes
13	62	Male	45	2	None	No	Inferior	Yes (n = 2)	3335	8.5	7.8	0.50	5.0	22	6	32	No
14	72	Male	27	9	Amiodarone	Yes	Apical	Yes (n = 5)	5353 9949∗	31.7 87.8∗	30.5 85.3∗	0.25 0.18∗	22.7 25.9∗	115	31	22	No
Total	63 ± 13	Male (86%)	35 ± 6%	5 (2–8.5)	—	No (79%)	—	Yes (79%)	4197 ± 2328	39.3 ± 22.9	36.6 ± 21.7	0.27 ± 0.08	16.6 ± 10.9	57.1 ± 8.8	25.0 ± 11.5	72.5 ± 28.4	No (93%)

Values are mean ± SD or median (interquartile range), unless otherwise indicated. Patients 2 and 11 had substrate maps collected in both atrial and ventricular pacing (top and bottom values in each column, respectively). Patients 1–9 did not have VT recurrence during follow-up; patients 10–13 had VT recurrence; patient 14 died.

AAD = antiarrhythmic drug; LVEF = left ventricular ejection fraction; VT = ventricular tachycardia.

∗ Epicardial maps.

### Omnipolar mapping acquisition

A total of 17 dense LV substrate maps (15 endocardial + 2 epicardial; 4197 ± 2328 points displayed) were collected either in atrial pacing (8 patients), right ventricular apical pacing (4 patients), or both (2 patients) per operator choice. In 5 patients, a hemodynamically tolerated VT was mappable. Tissue <0.50 mV was predominantly inferior (in 8 patients), lateral (1 patient), anteroseptal (1 patient), posterior (1 patient), and apical (3 patients), correlating with sites of wall motion abnormalities. We found omnipolar voltage area <0.50 mV to be significantly smaller than bipolar voltage area <0.50 mV (36.6 ± 21.7 cm^2^ vs 39.9 ± 22.2 cm^2^, *P* = .002).

### DVM analysis

The mean DVM-Th was 0.27 mV, though it spanned a wide range between 0.18 and 0.50 mV. Vector disarray on the DVM covered a significantly smaller shell area than the traditional 0.50-mV voltage threshold used to define scar (16.6 ± 10.9 cm^2^ vs 36.6 ± 21.7 cm^2^, *P* < .0001). The wide standard deviation in disarray area (ie, 10.9 cm^2^) reflected the observed variation between limited and extensive disarray. The percentage of vector uniformity was determined in different areas on the DVM, sampling myocardium covering 1 cm^2^. Tissue >0.50 mV demonstrated almost exclusively uniform vector activation (98.2 ± 2.7%), apart from in areas of wavefront collision, discernible on the corresponding vector propagation map. Tissue below 0.50 mV but above the DVM-Th revealed similarly high rates of uniform vector activation (97.5 ± 2.2%) (*P* = .32). In tissue below the DVM-Th, vector uniformity measured only 23.1 ± 5.3%.

**Figure 2:**
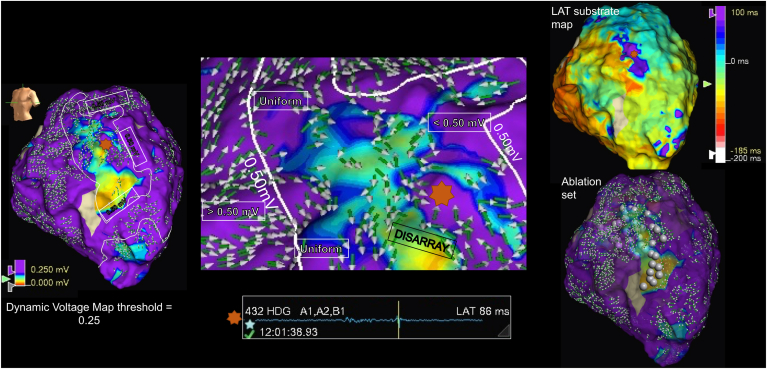
The dynamic voltage map vector disarray border zone colocates with areas of isochronal crowding (see also Video 2). (Far left) An atrial-paced voltage map of a postinfarct anteroseptal scar. The mid-anterior aspect appears uncolored, as data were not collected here owing to HD Grid instability. Omnipolar vectors have been superimposed onto the voltage map. A line was drawn around tissue with voltage <0.50 mV, and the voltage upper limit was sequentially reduced until the DVM threshold, here depicted at 0.25 mV. Orange star depicting an area of vector disarray. (Middle) Magnified view from the DVM, highlighting uniformity below 0.5 mV and disarray below the DVM threshold. The associated multicomponent late activating local electrogram from the orange star is highlighted beneath. (Right) Corresponding local activation time (LAT) map—the area of isochronal crowding coincided with the border zone of the DVM. Ablation set colocating within and around the DVM border zone.

**Figure 3:**
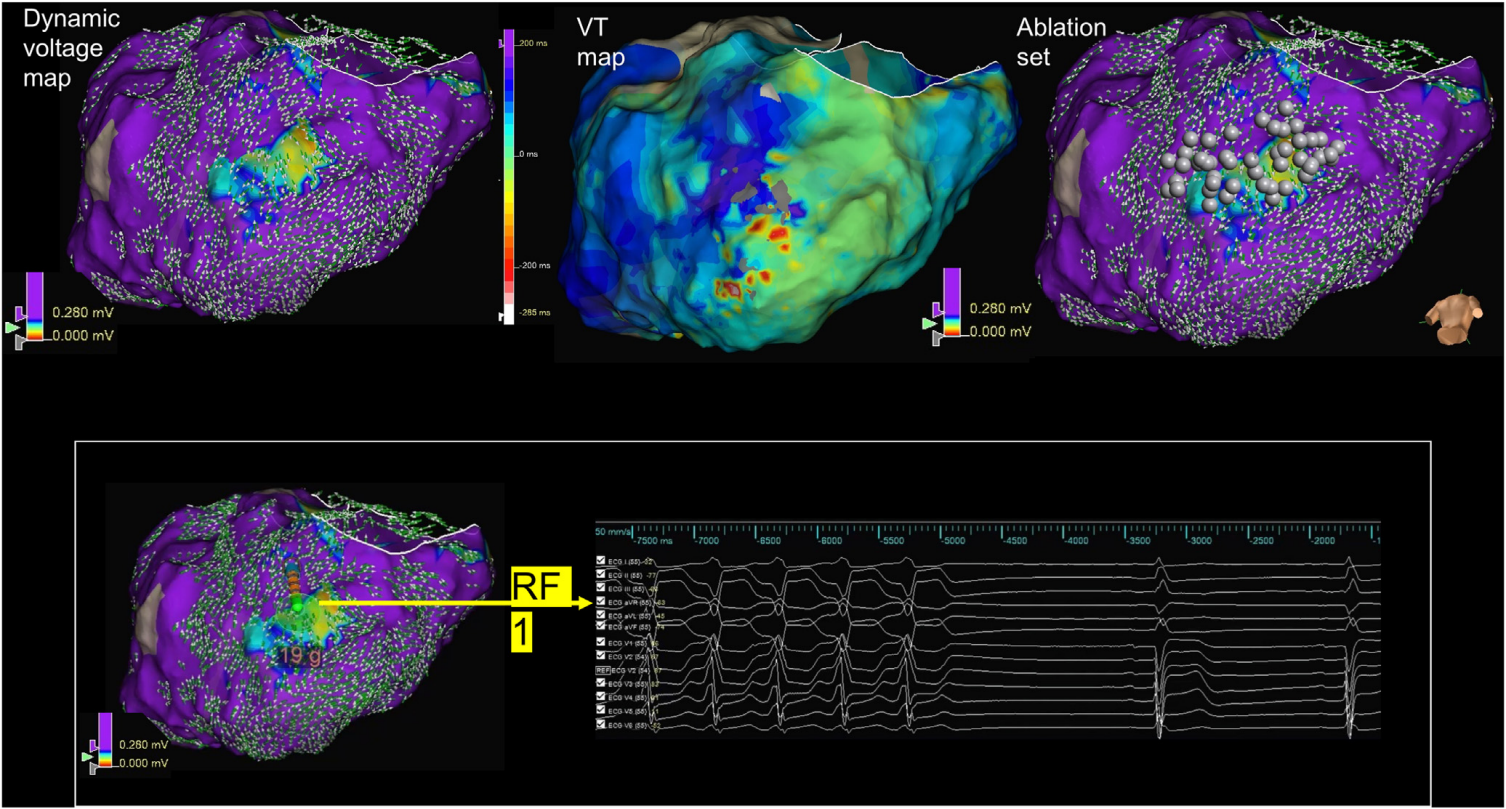
The dynamic voltage map (DVM) vector disarray border zone correlates with critical ventricular tachycardia (VT) components (see also Video 3). Patient with a postinfarct inferior scar. (Left, top) Right ventricular–paced omnipolar voltage map and omnipolar vectors demonstrating a DVM threshold of 0.28 mV. (Left, middle) Clinical VT activation map (cycle length 520 ms). Region bordering DVM threshold correlated with the diastolic pathway. (Top, right) Ablation set colocating with tissue within and around the DVM border zone. (Bottom) First application of radiofrequency (RF) resulted in termination of clinical VT.

**Figure 4:**
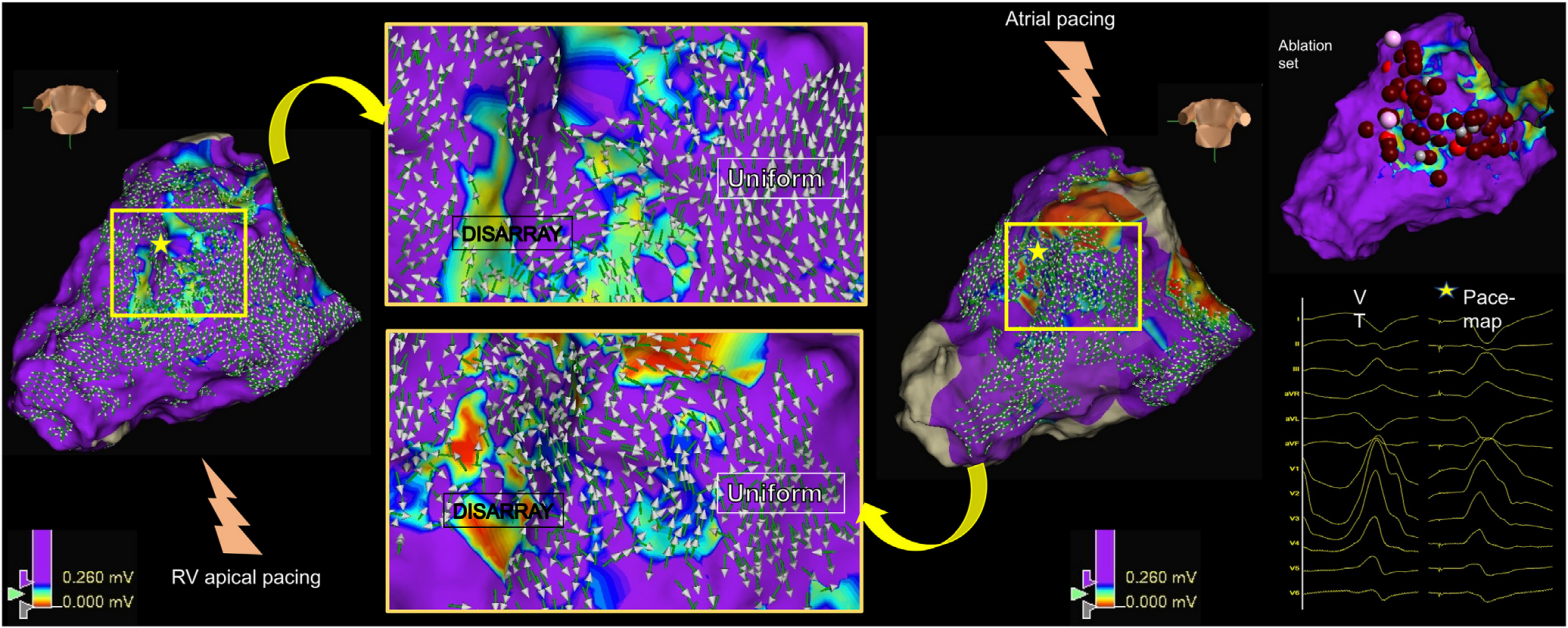
The dynamic voltage map (DVM) appears fixed despite changing pacing sites. (Left) Right ventricular (RV)–paced omnipolar voltage and omnipolar vector map in a patient with an inferior scar, adjusted to the DVM threshold (0.26 mV). (Center, top) Magnified area of RV-paced map to highlight vector disarray. (Right) Map from the same patient, collected in atrial pacing. The same DVM threshold (0.26 mV) was observed. (Center, bottom) Magnified area of atrial-paced map from the same location. Despite altering wavefront direction, the DVM appears generally concordant (with some minor discordance in areas of lower points density). (Far right, top) An excellent pace map morphology to the induced nonsustained ventricular tachycardia (VT) was observed in tissue near the DVM border zone (yellow star). (Far right, bottom) Ablation set colocating within and around DVM threshold.

As illustrated in Figure 2 (and Video 2), the area within the latest activation/site of isochronal crowding in a patient with prior anteroseptal infarct colocated along the DVM border zone, containing vectors transitioning from uniform to disarray. In Figures 1A, 1B, and 3 (and Videos 1 and 3), corresponding VT activation maps are compared with the DVMs. Components for the mapped VT isthmus also colocated along the DVM border zone. Figure 4 demonstrates a case of pace mapping along the DVM border zone, with excellent correlation to the induced VT. The substrate map in Figure 4 (and Video 4) was collected in 2 different paced wavefronts, and the DVM-Th was concordant between rhythms, consistent with a fixed postinfarct substrate.

There was a strong correlation between DVM-Th measurements between 2 observers (Spearman’s rho = 0.88, *P* < .0001). Bland-Altman plot showed good interobserver agreement, with a median difference of 0.00 mV, mean difference of –0.01 mV, and 95% limits of agreement from –0.04 to 0.01 mV.

A mean 57.1 ± 8.8 ablation lesions were delivered per patient (25.0 ± 11.5 minutes), and VT was noninducible in 13 (93%) patient’s postablation and antiarrhythmic medications were stopped. One patient died 2 months postablation due to heart failure progression. There was a significant reduction in VT burden (median 0 episodes, ranging from 0 to 4 episodes, *P* < .001), and 9 (69%) patients remained VT free at a median of 9 months. While there was no difference in the disarray area or number of ablation lesions between those with or without VT recurrence/death (16.9 ± 14.1 cm^2^ vs 14.2 ± 6.8 cm^2^, *P* = .70; 59 ± 24 lesions vs 54 ± 39 lesions, *P* = .80), the distribution of ablation lesions better colocated within or around the DVM border zone (ie, no more than 0.5 cm away) in those patients who remained VT free (86.5 ± 10.1 % vs 47.4 ± 34.1% of the total ablation lesions, *P* = .006). Video 3 reveals how the first ablation lesion that colocated to the DVM border zone terminated VT.

## Discussion

This study considers an alternative approach to analyzing a voltage map of postinfarct ventricular substrate using DVM in combination with omnipolar vectors. Our primary findings were (1) omnipolar vectors within post infarct scar can appear uniform or in disarray; (2) DVM revealed the mean voltage threshold differentiating uniform vectors from disarray was 0.27 mV, though ranging widely, suggesting that voltage thresholds are unique to each substrate; and (3) tissue within or bordering areas of vector disarray may harbor greater arrhythmogenic potential.

### DVM to help improve the utility of a voltage map

Voltage maps are often used to differentiate scar from border zone. However, the voltage limits that separate them are uncertain and probably vary between patients.^6^ Consequently, voltage-independent mapping techniques have become increasingly popular.^12^ In this study, we introduce DVM, which involves simultaneous appreciation of both activation and voltage maps. Our group recently studied ripple activation maps superimposed on a surface display of bipolar voltage on the CARTO system.^6^ Voltage settings were adjusted to distinguish tissue devoid of ripple bar activation, considered scar, from surrounding border zone tissue supporting ripple wavefronts. We have adapted this approach onto the EnSite X system using omnipolar vectors superimposed on a display of omnipolar voltage. The tissue with the greatest arrhythmogenic potential had numerically higher voltages using omnipolar mapping (ripple threshold 0.22 mV compared with 0.27 mV in this study), in keeping with both ours and other recent findings of higher voltages using omnipolar compared with bipolar mapping within postinfarct scar.^7^

### DVM guided by omnipolar vector disarray

Three-dimensional maps collected using bipolar EGMs on the EnSite X platform display the highest-amplitude EGM from 2 orthogonal HD grid signals. ^13^ Maps collected using Omni-EGMs use advanced signal processing to display the highest amplitude EGM of 3 adjacent HD Grid electrodes in 360°. While the utility of omnipolar voltage over bipolar voltage mapping has been described, omnipolar activation vectors have not been studied in detail beyond initial feasibility^14^ or local electrogram review.^15^ Preclinical canine omnipolar studies in atrial fibrillation considered areas of vector disorganization in areas of low voltage as potential targets for substrate ablation.^16^ In a study specifically describing omnipolar vectors involving human atrial tissue, the authors questioned the utility of vectors in low-voltage myocardium, speculating that this may reduce reliability of global activation interpretation.^17^ While this is certainly true, the scarred arrhythmic ventricular substrate is not organized, and we have considered the observed vector disarray a surrogate for the chaotic wavefront turning and collision in and around fixed myocardial scar. In this study, we found that tissue bordering areas of vector disarray (ie, the DVM border zone) colocated with sites of latest activation/isochronal crowding; as well as critical components of the mapped VT and excellent VT pace map correlations.

Time-dependent mapping has well-known limitations, especially in areas of low voltage.^18^ Omnipolar vectors obviate the need to assign a fiducial reference or select a single activation time within a window of interest to often multicomponent EGMs (which can require retrospective manual reannotation). Furthermore, the local activation time (LAT) of a multicomponent EGM is interpolated by the LAT of its neighbor, whereas an omnipolar vector is not influenced by its neighboring vector; hence, rogue signals may have less impact on the omnipolar vector map.^11^ While LAT propagation maps attempt to create a comprehensible best fit pattern of activation within low-voltage tissue, omnipolar vectors may depict their localized anisotropic zigzag conduction as disarray.^19^

### DVM-Ths are individualized

The DVM-Th and area of vector disarray varied between patients, as was seen in our previous work with ripple mapping,^6^ suggesting that empirical voltage cutoffs (eg, 0.20 mV–0.50 mV) may be misleading, as they fail to account for individual patient variation. Importantly, despite marked variation in DVM-Th between patients, blinded interobserver agreement was high in individual cases. The reason for interpatient variations is unknown and may be consequent to the underlying tissue thickness and scar construct.^20^ We speculate that those with limited disarray represent a single line of conduction slowing/block and that those with extensive disarray (exemplified in Figures 1 and 4) reflect multiple lines of conduction slowing/block.

### DVM as a potential guide to substrate ablation

As illustrated in all our figures, components of the VT isthmus/best pace-maps/isochronal crowding/late activation all colocated within the border of purple to nonpurple tissue containing vectors transitioning from uniform to disarray along the DVM border zone. Furthermore, we observed that ablation better colocated around the DVM border zone in patients without VT recurrence during follow-up. These findings suggest that tissue near the DVM border zone may harbor higher arrhythmogenic substrate. We hypothesize that targeted ablation in and around the DVM border zone may result in high rates of freedom from VT recurrence without the need for extensive scar homogenization, though this requires prospective testing.

Ablation was also observed within areas of vector disarray. The recent appreciation of the 3-dimensional postinfarct electroarchitecture has highlighted the extent of layered VT substrate, beyond the mapped surface.^21^ Indeed, the mapped VT in Figure 1 suggests a part-epicardial circuit owing to an activation timing gap in the endocardial map. In such a case, ablation within disarrayed tissue may still be required to treat deeper tissue that might support uniform vector activation within intramural or epicardial layers.

### Limitations

This study was a single-center retrospective analysis of a small group of patients; however, our primary purpose was not to present ablation outcome data, but rather to describe a simple, practical, and reproducible mapping technique, readily available on the EnSite X system, to help improve the clinical utility of a voltage map during VT ablation.

The interpretation of disarray can be subjective, based on the general profiles of vector orientations. Furthermore, as with other substrate mapping modalities, it is challenged in areas of lower point density, which can occur in areas harder to map with a grid-like catheter, such as under the mitral valve leaflets, as exemplified in Figure 4.^10^ However, we found strong interobserver agreement of DVM-Th measurements between blinded independent operators.

**Figure 5:**
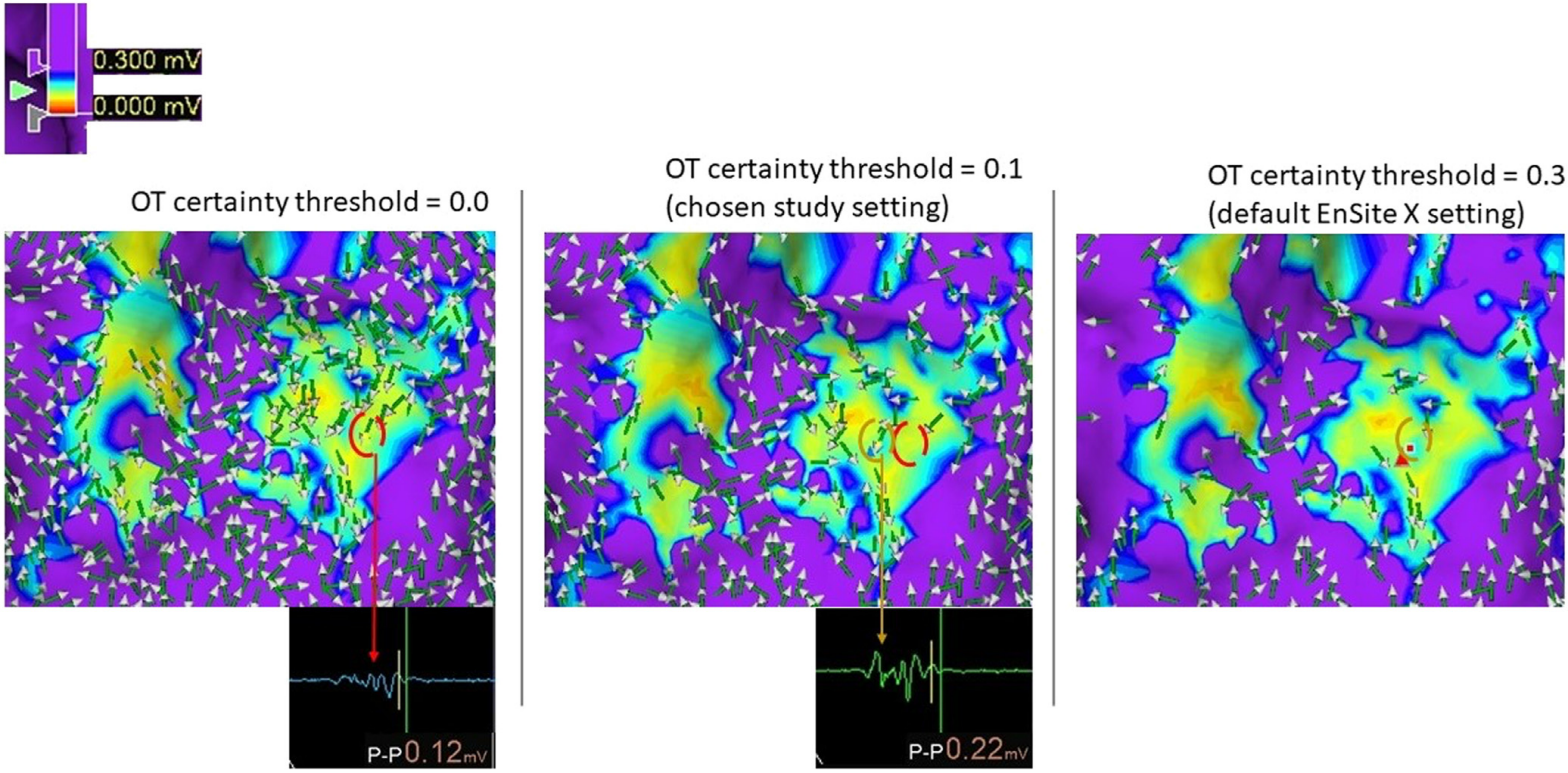
Omnipolar Technology (OT) certainty threshold. The 3 panels demonstrate the same magnified image of an area of low-voltage tissue <0.30 mV. Omnipolar vectors have been superimposed. In each panel, the applied OT certainty threshold has been adjusted (left: 0.0; middle: 0.1; right: 0.3). With increasing OT certainty threshold from left to right, the number of arrows displayed can be seen to reduce. The panel on the right with OT certainty threshold of 0.3 represents the default system setting, in which the assessment of vector disarray is limited by low arrow density and hence was not favored in the study. An arrow is encircled red in the left panel. With increasing OT certainty from 0.0 to 0.1, the same arrow cannot be seen in the middle panel. The corresponding omnipolar electrogram associated with this arrow vector is displayed with peak-peak (P-P) voltage 0.12 mV. Another arrow is encircled light brown in the middle panel. With increasing OT certainty from 0.1 to 0.3, the same arrow cannot be seen in the right panel. The corresponding omnipolar electrogram associated with this arrow vector is displayed with P-P voltage 0.22 mV. Applying the default OT certainty threshold of 0.3 would remove this important multicomponent fractionated electrogram from the map. Even though some vectors with associated very low-voltage electrograms (eg, 0.12 mV) arise only at a threshold of 0.0, we chose not to include 0.0 as the study setting, as too low a value could risk the incorporation of noise; hence, we accepted the 0.1 OT certainty threshold as the study setting.

We chose an OT certainty threshold of 0.1 to maximize the number of vectors on display, and excluded vectors associated with the greatest degree of EGM uncertainty that might remain at 0.0. However, as demonstrated in Figure 5, some vectors associated with very low voltage (eg, 0.12 mV) true EGMs may have been removed from the display. The algorithm is industry proprietary and otherwise unstudied, thus it remains unclear what effect various settings will have. Vector disarray can also be seen in healthy myocardium and can be misleading when adopting the DVM approach. This occurred for 2 reasons: (1) where 2 passive wavefronts travelling across the myocardium collide, or (2) in sites of activation origin into the left ventricle, and these were obvious on the corresponding vector propagation map.

While the association between freedom from arrythmia recurrence and greater DVM border zone ablation is interesting, this requires prospective study to determine its value beyond established strategies. No entrainment mapping was used to verify the putative critical isthmus sites. The incremental utility of vector disarray independent of other means of substrate characterization (fragmentation, late activation, and capture with latency, etc.) remains to be determined. However, even without this, vector disarray facilitates the DVM technique, which may provide helpful corroboration when conventional approaches are less forthcoming.

## Conclusion

We describe a practical approach to analyzing a voltage map of the postinfarct ventricular substrate using DVM in combination with omnipolar technology on the EnSite X platform. Omnipolar vectors within postinfarct scar can appear uniform or in disarray, with differentiating voltage thresholds unique to each scar. Tissue bordering areas of vector disarray may harbor greater arrhythmogenic potential.

## Disclosures

Nicole Worthington and Calum Phenton are employees of Abbott Medical and offered manuscript quality assurance. All other authors declare no conflicts of interest relevant to this study.

## Appendix Supplementary Data

Supplementary video 1 **Dynamic Voltage Mapping guided by Omnipolar Vector Disarray.** Right ventricular-paced Omni-voltage map of a left ventricular inferior wall scar displayed between 0.0 mV - 1.50 mV scale. Omni-vectors have been superimposed onto the voltage map. The upper voltage limit was subsequently adjusted to 0.50 mV and a line was drawn around tissue with voltage bordering 0.50 mV. The voltage upper limit was sequentially reduced until only those areas with vector disarray were observed below it (a zoomed in version is depicted in Figure 1b). This voltage cut-off, termed the Dynamic Voltage Map threshold (DVM-Th), here is 0.20mV. Monomorphic VT was mapped using LAT – whilst the full circuit was not contained within the endocardial surface (evident by a colour timing gap), components of the VT isthmus (red colour) correlated with tissue bordering the DVM-Th. *Right (bottom)*: Ablation set co-locating within and around the borderzone of the DVM. mV – millivolt; VT – ventricular tachycardia.

Supplementary video 2 **Dynamic Voltage Map Vector Disarray borderzone co-locates with areas of isochronal crowding.** An atrial paced voltage map of a post infarct anteroseptal scar. The mid-anterior aspect appears un-coloured as data was not collected here owing to HD grid instability. Omni-vectors have been superimposed onto the voltage map. A line was drawn around tissue with voltage <0.50 mV and voltage upper limit was sequentially reduced until DVM-Th, here depicted at 0.25 mV. Orange star depicting an area of vector disarray. Magnified view from the Dynamic Voltage Map, highlighting uniformity below 0.5 mV and disarray below the DVM-Th. The associated multicomponent late activating local EGM from the orange star is highlighted beneath. Corresponding LAT map – the area of isochronal crowding coincided with the borderzone of the DVM. Ablation set co-locating within and around the DVM Borderzone. DVM-Th – Dynamic Voltage Map threshold; mV – millivolt; LAT – local activation time, EGM – electrogram; VT – ventricular tachycardia.

Supplementary video 3 **Dynamic Voltage Map vector disarray borderzone correlates with critical VT components.** Patient with a post infarct inferior scar. Right ventricular-paced Omni-voltage map and Omni-vectors demonstrating a DVM-Th of 0.28 mV. Clinical VT activation map (cycle length 520ms). Region bordering DVM-Th correlated with the diastolic pathway. Ablation set co-locating with tissue within and around the DVM borderzone. First application of RF resulted in termination of clinical VT. VT – ventricular tachycardia; RF – radiofrequency.

Supplementary video 4 **Dynamic voltage map appears fixed despite changing pacing sites.** RV-paced Omni-voltage and Omni-vector map in a patient with an inferior scar, adjusted to the DVM-Th (0.26 mV). Magnified area of RV paced map to highlight vector disarray. Map from the same patient, collected in atrial pacing. The same DVM-Th (0.26 mV) was observed. Magnified area of atrial paced map from the same location. Despite altering wavefront direction, the DVM appears generally concordant (with some minor discordance in areas of lower points density). An excellent Pace-map morphology to the induced non-sustained VT was observed in tissue near the DVM borderzone (yellow star). Ablation set co-locating within and around DVM-Th. RV – right ventricle; mV – millivolt; VT – ventricular tachycardia.
